# Clinical Genome Data Model (cGDM) provides Interactive Clinical Decision Support for Precision Medicine

**DOI:** 10.1038/s41598-020-58088-2

**Published:** 2020-01-29

**Authors:** Hyo Jung Kim, Hyeong Joon Kim, Yoomi Park, Woo Seung Lee, Younggyun Lim, Ju Han Kim

**Affiliations:** Seoul National University Biomedical Informatics (SNUBI), Division of Biomedical Informatics, Seoul National University College of Medicine, Seoul, Republic of Korea

**Keywords:** Data integration, Health care, Medical research, Translational research

## Abstract

In light of recent developments in genomic technology and the rapid accumulation of genomic information, a major transition toward precision medicine is anticipated. However, the clinical applications of genomic information remain limited. This lag can be attributed to several complex factors, including the knowledge gap between medical experts and bioinformaticians, the distance between bioinformatics workflows and clinical practice, and the unique characteristics of genomic data, which can make interpretation difficult. Here we present a novel genomic data model that allows for more interactive support in clinical decision-making. Informational modelling was used as a basis to design a communication scheme between sophisticated bioinformatics predictions and the representative data relevant to a clinical decision. This study was conducted by a multidisciplinary working group who carried out clinico-genomic workflow analysis and attribute extraction, through Failure Mode and Effects Analysis (FMEA). Based on those results, a clinical genome data model (cGDM) was developed with 8 entities and 46 attributes. The cGDM integrates reliability-related factors that enable clinicians to access the reliability problem of each individual genetic test result as clinical evidence. The proposed cGDM provides a data-layer infrastructure supporting the intellectual interplay between medical experts and informed decision-making.

## Introduction

As the field of medicine transitions from experience-based medicine to data-driven medicine, an apparent paradigm shift to precision medicine is underway, driven by the development of technologies in fields including medical information technology and computer engineering^1,2^. Genomic information is one of the most critical components of precision medicine, given its power to explain individual variability^3^. However, the practical clinical use of genomic information remains limited because its circulation is suboptimal, with each data processing step tending to be independently performed and thus isolated. To narrow this gap, many organisations have attempted to identify and develop methods to more effectively link genomic data to clinical information and thereby facilitate its use^4–6^. However, several challenges must be surmounted before realising this goal.

First, a mismatch exists between the structure of genomic and clinical data. Genomic data based on next-generation sequencing (NGS) technology is stored as a number of file types at various stages of the bioinformatics analysis, with flexible file specifications to accommodate the broad range of research interests in bioinformatics^7^. Raw genomic data can contain up to several tens of gigabytes of sequence information, each stored as a long string of data, and therefore cannot be used directly in this form in clinical practice without further processing. Since data processing to determine clinical relevance is both computationally intensive and time-consuming, genomic information is not readily accessible relative to other types of clinical data. Thus, for precision medicine and personalised medicine, pre-processed genomic data need to be linked with other clinical information and provided at the appropriate time. To resolve this issue, a structured database is needed to store and appropriately manage genomic information for easy accessibility.

Second, genomic data have different properties than conventional observational data used in clinical settings. Therefore, genomic data must be clarified by considering procedural dimensions. Since genomic workflows contain a large number of pipelines for information processing, significant differences between the interpretation of processed data and data obtained from different information systems relative to the clinical workflow is inevitable^8^. Accordingly, a robust data model is required to serve as an information system to systematically manage genomic data, encompassing the detailed processes of data processing, analysis, and filtering. Additionally, information on the reliability and accuracy of these analyses results, along with the detailed analytical process and equipment used, must also be systematically stored and managed, as it is an essential criterion for clinical decision-making^9^. Moreover, because genomic data is less variable than observational data, information integration will allow for maximisation of the utility of the collected genomic information for clinical use.

The third challenge majorly hindering the integration of genomic data with clinical information is difficulty in mapping the two types of data for medical interpretation. The presence of biomarkers for specific diseases or drug reactions is a critical factor in clinical decision-making^10^. In the case of targeted sequencing, the data processor is informed about biomarkers related to the panel prior to analysis. In clinical practice, reannotation of patient genetic information according to updated biomarker discoveries from the biomedical research community is continuously required at the population level. Thus, a structured data model with consistent data representation would enable the rapid adoption of both evolving biomedical knowledge and individual medical records, which can be delivered to the point of care through agile data processing. Furthermore, patient genomic data expressing specific biomarkers should be readily accessible from the information system along with clinician-confirmed interpretations^11^.

**Figure 1:**
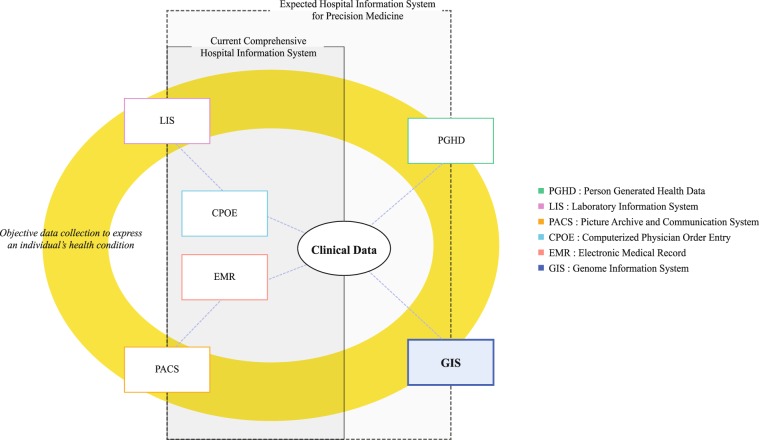
Data-level linkage structure between conventional HIS and GIS. From a software engineering perspective, a comprehensive hospital information system comprises components that represent separated data collection routes and distinguishing characters of the data. We suggest the concept of GIS to illustrate the implementation of the cGDM. This architecture supports both information and functional integration, even with existing clinical information systems.

Personal-health status can be converted to a composition of multi-layered, multi-dimensional digitalised information for utilisation in an information system that facilitates handling big data (Fig. 1). Indeed, vast amounts of data and associated metadata from multiple medical measuring technologies, such as laboratory tests or imaging studies, have already been successfully merged in clinical information systems. Overall, although genomic information represents the most sound and intensive health-related signals provided by the human body throughout life, the weak links to medical practice highlighted above contribute to its underutilisation in clinical decision-making. Therefore, it is necessary to effectively link and integrate clinical information with personal genomic information, helping to accelerate the shift to personalised medicine.

Toward this goal, we aimed to develop a clinical genome data model allowing for enhanced interactive support in clinical decision-making, which minimises the possibility of misinterpretation at the point of care, due to formal and procedural heterogeneity related to the production process. We began by redefining the obstacles to the spread of genomic information into routine care, including problems relating to the reliability of measurements that could cause hesitation in clinical decision-making and data structural problems that have hindered integration of genomic data into existing information systems. From a clinical perspective, we focussed on clarifying not only the problem of heterogeneous data structure issues but also reliability-related factors. In this context, we operationally defined a bioinformatics process not as a “measurement”, but rather as a “production” requiring transition a physical form of existence to a human-interpretable representation. Thus, informational modelling based on workflow analysis was used as a ground knowledge for a communication scheme between sophisticated bioinformatics products and a representative component of data, which is essential for a proper clinical decision.

## Results

This section primarily consists of Failure Mode and Effects Analysis (FMEA) results and entity-attribute modelling. FMEA output is presented in two diagrams: a dataflow diagram that focusses on the derivation of the contents of the genetic test based on NGS sequencing technology, and an information process map that extends the viewpoint to the level of clinico-genomic context. At this step, the protocol entity of the former dataflow diagram was subclassified to reveal the procedural dimension in information processing. Moreover, the set of attributes involved in each step of information transfer was identified. Finally, the cGDM are suggested as a result of structured data modelling based on the attribute set.

### Dataflow diagram based on an NGS workflow

**Figure 2:**
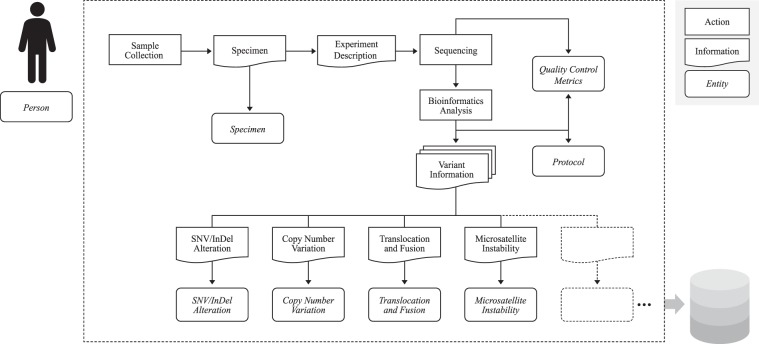
Data flowchart based on a next-generation sequencing workflow. The objects shown in this diagram are classified into three class types- **‘**Action**’**, **‘**Information**’**, and **‘**Entity**’**. ‘Action’ was first posted with respect to what occurred in each expert domain and the resulting ‘Information’ was displayed as a result of each action. Finally, **‘**Entity**’** was defined as the captured information class at each stage of the workflow. Subtypes of **‘**Variant Information**’** were drawn scalable to accommodate the potential extension of subclasses.

A workflow diagram was derived in order to illustrate the data flow in which the genomic information inherent in the human body is converted to a genomic test result. (Fig. 2.) At this stage, the clinical view is minimised, with both the flow of information and the process of analysing the specimen after the sample collection across experimental laboratory and computational analysis drawn on a large scale.

The subtypes of processed variant information in the parallel structure, used to cope with the growing body of knowledge in bioinformatics, are listed at the bottom of Fig. 2. Variant information can be called in multiple types depending on the perspective and purpose of the analysis. For example, there are four types of genetic variation: single nucleotide variation (SNV), small insertion/deletion (InDel), copy number variation (CNV), and translocation/fusion. There are predictive biomarkers as well such as microsatellite instability (MSI) and tumour mutation burden (TMB). As the amount of NGS technology-based knowledge increases, more subclasses representing novel perspectives can be added. Scalable data modelling to support the differentiation of knowledge over time is essential not only for expressiveness, but also for reducing the burden of information systems maintenance.

In summary, we linked the separate offline workflows at this step that occurred in different places until genomic data could be provided as processed data. The workflow diagram provided the basis for detailed analysis and discussion.

### Extending the NGS process under a clinico-genomic context

After establishing consensus on a larger scale, we extended the flow of information to the clinical context in detail. At this stage, the standpoint of the workflow analysis was clinical decision making. Hence, the workflow diagram started with a clinical decision. We extended the flow between several actions in the clinico-genomic context involving multiple entities identified, and detailed analysis was performed. In this process, the output data file format and detailed processes for handling output files, along with the tools required for linking to external knowledge databases, are also described.

**Figure 3:**
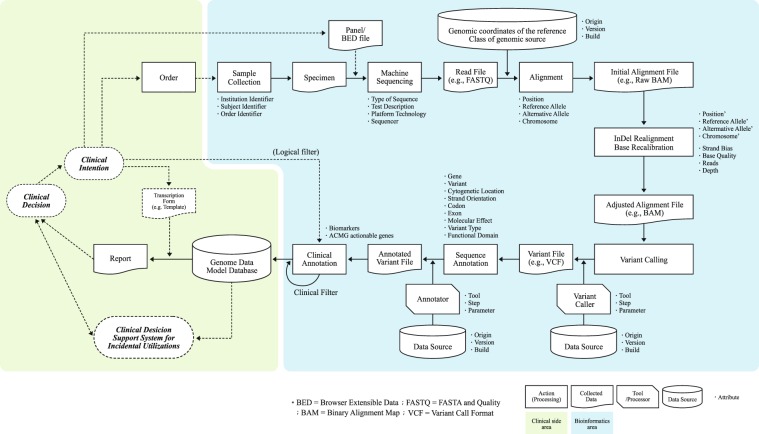
Failure mode identification: mapped next-generation sequencing process extended to a clinico-genomic context. In the bioinformatics area (cyan background), information may be distorted by the insufficient representation of origin, processing rule, and external reference. To prevent this failure, identification and semantics, related attributes are listed under the boxes. In the clinical area (yellow background), the data model functions as a communication scheme for the collaborative process implemented in the hospital information system. Data-level integration facilitates just-in-time queries and reuse of data.

The working group discussed mechanisms for extraction of the entity-attribute set which would avoid probable information distortion and omission. We considered that the genomic data model for clinical use should be the knowledge communication scheme, thus preserving its reliability-related factors. At a minimum, the genomic data model must provide sufficient information to decide whether the confidence level of the genomic test result justifies its consideration as clinical evidence. For this function, failure was defined as that which causes misinterpretation or non-use of the genomic data for clinical decision. The process of producing clinical evidence from genomic data at the bioinformatics area (Fig. 3) shows a pattern that is a series of repeated representations of information converted by reference knowledge bases and data processing rules. Thus, failure modes can be classified as incomplete specifications in three meta-categories: origin, reference, or symbol. Due to the nature of the semantic interpretation, any fragmentation of symbol causes not only loss of information but also assignment information to direct the origin^12,13^.

We conducted workflow analysis to extrapolate general descriptors of the related attributes with the goal of preserving information during production and delivery processes from clinical intention to clinical utilisation. Figure 3 provides a more detailed data-level view, including how genomic information is realised as clinical evidence in a case based on a structured data model. The structured genome data model can support a report via presentation on a variety of transcription forms (report forms), which are optimised for initial intent. Furthermore, additional utilisation paths are accessible in the clinical-information system. As shown in Fig. 3, data-level integration helps the amplification of the incidental utilisation. (Supplementary Fig. 3) To illustrate, consider a patient who orders whole-genome sequencing to screen for cancer biomarkers at their first visit. When the patient receives a prescription for antibiotics a year later at a visit for other symptoms, that same genomic test result can be re-used from a pharmacogenomics perspective for safer and more efficient drug prescription. The clinical decision support system plays a vital role by just-in-time display of the matching information with pre-defined rule and knowledge-based processing^6,14,15^. A computational genome data model is a prerequisite for this implementation^15–17^. Finally, we introduce a logical data model in the next step of the study.

### The cGDM

#### Logical data structuring with the entity-attribute model

**Figure 4:**
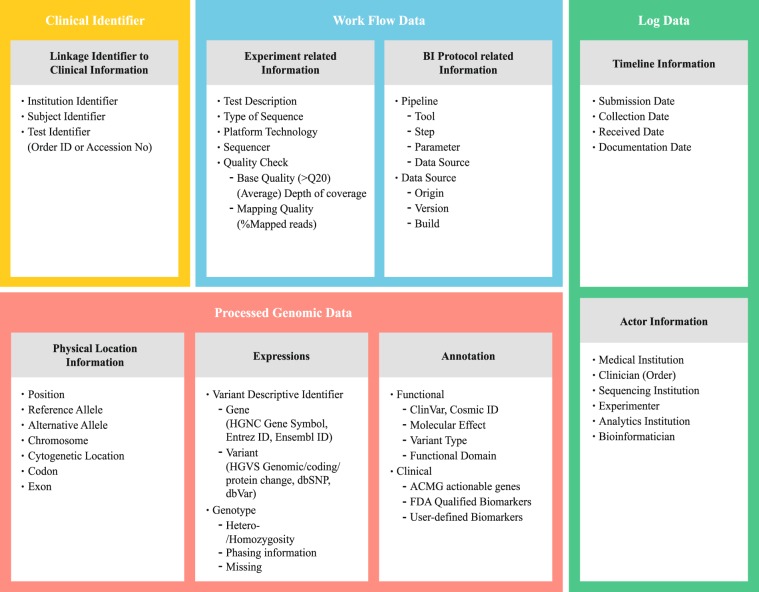
The Clinical Genome Data Model: Structured data modelling with entities and attributes. The cGDM is designed as a logical data model of 8 entities and 46 attributes. The objects and related attributes derived through FMEA are integrated into a logical data model through abstraction and normalisation.

Finally, the cGDM was designed as an entity-attribute model consisting of 8 entities and 46 attributes (Fig. 4). For a structured data model of the identified clinico-genomic attributes, logical modelling was conducted to ensure data-level linkage with conventional primary clinical databases. In order to define the entity-attribute model based on the action and collected data, tool/processor classes and the attributes of each class from Fig. 3, we define 3 types of classes as protocol and related attributes (Table 1). Since the cGDM is designed to support data-level integration with the existing system, only the minimum subject identifier is defined as ‘linkage identifier to clinical information’. To represent the procedural dimension, which is stressed in the study, we combined two workflow analyses on different scales. For example, the entity ‘Protocol’ as a part of the procedural dimension is explicitly represented in Fig. 2, then expressed again as a list of lower steps in Fig. 3. Since clinical observation is typically considered as the collection of events^18^, the logical composition of the date/time and actor identifier related to the clinico-genomic context were declared.

**Table 1:** Extracted classes and related attribute sets from each step of clinico-genomic context for the Entity-Attribute model.

Seq.	Class			Related Attribute	Entity
	Action	Collected Data	Tool/Processor		
1	Sample Collection			Institution Identifier Subject Identifier Test Identifier (Order ID or Accession No)	Linkage Identifier to Clinical Information
				Submission Date	Timeline Information
				Medical Institution	Actor Information
				Clinician	
2		Specimen			
3	Machine Sequencing			Test Description	Experiment Related Information
				Type of Sequence	
				Platform technology	
				Sequencer	
				Collection Date	Timeline Information
				Sequencing Institution	Actor Information
				Experimenter	
4		Read File			
5	Alignment			Position Reference allele Alternative allele Chromosome	Physical(Location) information according to coordinate system
				Analytics Institution	Actor Information
				Bioinformatician	
6		Initial Alignment File			
7	InDel Realignment			Position’	Physical(Location) information according to coordinate system
	/			Reference allele’	
	Base Recalibration			Alternative allele’	
				Chromosome’	
				Base quality(>Q20)	Quality Check information
				(Average) Depth of coverage	
				Mapping Quality (%Mapped reads)	
				Received Date	Timeline Information
				Analytics Institution	Actor Information
				Bioinformatician	
8		Adjusted Alignment File			
9	Variant Calling			Hetero-/Homozygosity	Genotype Expressions
				Phasing information	
				Missing	
				Analytics Institution	Actor Information
				Bioinformatician	
10			Variant Caller	Tool	Pipeline information
				Step	
				Parameter	
				Origin	Data source
				Version	
				Build	
				Parameter	
11		Variant File			
12	Sequence Annotation			Gene (HGNC Gene Symbol, Entrez ID, Ensembl ID)	Variant Descriptive Expressions
				Variant (HGVS(genomic, coding, protein change + version), dbSNP, dbVar)	
				Cytogenetic location	Physical(Location) information according to coordinate system
				Codon	
				Exon	
				ClinVar, COSMIC ID	Functional Annotation
				Molecular Effect	
				Variant Type	
				Functional Domain	
				Analytics Institution	Actor Information
				Bioinformatician	
13			Annotator	Tool	Pipeline information
				Step	
				Parameter	
				Origin	Data source
				Version	
				Build	
14		Annotated Variant File			
15	Clinical Annotation			ACMG actionable genes	Clinical Annotation
				FDA qualified biomarkers	
				User-defined biomarkers	
				Analytics Institution	Actor Information
				Bioinformatician	
				Documentation Date	Timeline Information

The processes in the clinico-genomic workflow shown in Fig. 2 are listed in order and associated with the classes, related attribute sets for each process. This table is an intermediate result between the result of FMEA and the final logical model. Derived related attributes are abstracted within each class and grouped into entities.

The derived classes and entities in Table 1 were used to declare final entities and attributes in the cGDM (Fig. 4). The mapped Actions and Action-related classes (Collected Data and Tool/Processor) are categorized into subdomains and related attributes for each step in Table 1. In Table 1, an action and its result are grouped into one step, and the related attributes are represented by the attributes classified in the corresponding step. For normalization, related attributes are categorized to create one or more new groups called entities for each step, and they are the basis for defining ‘Entities’ in the Entity-Attribute model (Fig. 4). For example, ‘Physical information according to coordinate system’ is one of the three subdomains of the action ‘Sequence Annotation’. It can include an attribute set include an attribute set (Cytogenic location, Codon, Exon) representing physical location information for each variant. However, this “Physical information according to coordinate system” can be a subdomain in other steps besides “Sequence Annotation”. And even though it is the same subdomain, the related-attribute set may be different depending on which step or action. In summary, each step identified in the entire clinico-genomic process can include multiple entities, and one entity can be related to multiple steps. Even in the same entity, the configuration of related attribute as a factor affecting each step may vary from step to step.

#### Enhancing the reliability of genomic data by the cGDM

We conducted additional analyses to evaluate whether the cGDM can represent sufficient information scale to access the reliability of delivered information. We classified three selected examples of errors that often occur in genomic data processing into two distinct types: insufficient information scales and multiple names for the same variant. We then checked whether process errors of these types could be covered by the cGDM.

#### Insufficient information scale to detect clinically significant genetic variation

In this category, we discuss two typical errors: the absence of RefSeq accessions and versions in Human Genome Variation Society (HGVS) nomenclature, and nonexistence in human genome reference assembly. Both are cases in which the information scale is insufficient for the detection of clinically significant genetic variations.

Refseq accessions and versions for HGVS nomenclature. In the first example, a genetic biomarker has the potential to be clinically utilised for diagnosis, prognostics, and prevention^19,20^. The conventional way to represent genetic biomarkers is by protein-level HGVS nomenclature, which describes amino-acid sequence changes^21,22^. According to standard nomenclature recommendations of the HGVS, a RefSeq accession and version number are required, followed by information on amino-acid sequence changes. However, since the expression has usually been used without the accompanying reference sequence information upon which the numbering system is defined, this incomplete representation leads to a misinterpretation of the results for use in real clinical settings. For example, the BRAF V600E mutation is the most common driver in melanoma^23^. This BRAF mutation is represented as ‘NP_004324.2:p.V600E’ with the corrected version of the nucleotide sequence, but was formerly expressed as ‘NP_004324:p.V599E’ based on the nucleotide sequence missing a codon in exon 1^24^. Because V600E is recognised as a biomarker in the majority of publications, V599E without reference sequence information has been detected as a distinct mutation. To reduce such discrepancies, the proposed cGDM implements a more complete set of elements: the RefSeq accession, version number, and amino acid changes. Since the purpose of the cGDM is to eliminate ambiguity in information delivery, the set of attributes needed to point out a particular mutation is declared. Thus, the cGDM enables more reliable query result suggestions, even when the inputted search term is fragmented. Supplementary Figure 2 demonstrates a scenario in which a clinician performs a semantic query on a melanoma patient in a cGDM system. Importantly, the cGDM links the extracted annotation information to the results that the clinician would like to retrieve.

Human genome reference assembly. In the second example, the most popular way to determine genetic variants in standard NGS analyses is resequencing, which identifies variations by aligning reads against the reference genome sequence^25,26^. However, this often causes assembly errors. Since a variant position designates a relative location based on a given reference sequence, the data sources to which the reads are aligned are necessarily required. For example, the genomic position of BRAF V600E, chr7:140453136 A > T (GRCh37), is shifted into chr7:140753336 A > T (GRCh38) as that the significant reference coordinate has changed. Therefore, the cGDM is designed to specify the reference data sources, including assembly, version, and origin, greatly reducing reference compatibility problems induced by fragmented information.

#### Unnormalised representation of genetic variants

Non-unique expression of insertion and deletion. The second main error type involves the standardised representation of genetic variants, for which we exemplify issues arising due to the multiple names associated with the same insertions and deletions in databases. Variant Call Format (VCF) is a file format that allows for a flexible representation of different types of variations. Since each variant caller reports in a slightly different manner, the same variant could be represented in non-unique ways^27^. These inconsistencies across tools hamper the robust identification of clinically significant variants^28^. Thus, a specification for the unified representation of genetic variants is in high demand. For example, chr10:11805838 C > CT and chr10:11805838 CG > CTG represent the same insertion, even though they are not represented by the same text string. To overcome the issue, when importing VCFs into the cGDM, the representation of variants is converted into their minimal representation via tools such as *vcflib vcfallelelicprititives* or *GATK LeftAlignAndTrimVariants*. This process establishes consistency between internal and external representations and provides a standardised variant representation to ensure accurate and consistent identification of clinically significant variants.

The two types of problems described above can be solved using the entities and attributes defined in the cGDM. In solving the first problem of an insufficient information scale, the primary challenge is securing an element set that can convey the complete sense when expressing specific information. In the cGDM, since all factors causing this problem are represented by an entity-attribute set, no loss of information occurs. In the second type of problem, our example highlighted that models can be derived from the same semantics despite different nomenclature (syntax) in the stored values.

Detailed considerations made in this section highlight the differences in perspectives between the bioinformaticians and clinicians participating in the working group on the clinico-genomics workflow. The cGDM was placed in this interspace for systematic information management, with the application of reliability engineering to reduce miscommunication and distortion arising from a difference of viewpoint between experts in different fields.

### Validation of the cGDM

Here, the cGDM was finalized in the form of a logical model, which allows adaptation to the diverse development environments of existing heterogeneous clinical information systems. Logical model can play an essential role to generalize the complex phenomenon by abstraction and enhance understanding core ideas the model deliver between different stakeholders of in the complex system^29^. Whereas, the drawback of this approach is that physical modelling layer is needed in order to the data model implementation and validation. Thus, we design a physical data model implemented in a relational database to evaluate the model validity for real-world data and to proof of concept how implementation of the cGDM enables interactive clinical decision support in clinical information system shown as Fig. 3 (Left side; Clinical decision support system for incidental utilization).

#### Implementation of the real world data

This physical data model of the cGDM is provided in forms of entity-relationship diagram and table (Supplementary Information Table 1; Fig. 2). Also, one-click executable data definition language script is also freely accessible on a web page (https://github.com/SNUBI-HyojungKim/cGDM-Clinical-Genome-Data-Model).

For the data model validation with real-world data, we built pilot databases based on the cGDM and uploaded genomic data of over 2,000 patients for multiple diseases, including acute lymphoblastic leukemia, solid cancers, and depression cases (Table 2, internal databases). However, the pilot datasets related researches remain undergoing, we have built two representative demo datasets for open source (Table 2, demo databases) 1000 genome CEU (Utah Residents with Northern and Western European Ancestry) population dataset for whole genome sequencing (n = 99, 47.67 GB), 2) TCGA PAAD (Pancreatic Adenocarcinoma) dataset for somatic mutation (n = 155, 9.41 MB). We believe those well-known public dataset have advantages on data validation issue. Every demo datasets and source codes are freely available from at the Github page as mentioned above.

**Table 2:** Summary of imported genomic data from various data sources in cGDM databases.

	Table name	Database						
		Internal database					Demo database	
		Cancer Panel	Leukemia	Depression	TCGA COAD	TCGA LUAD	1000 Genome Phase 3 CEU	TCGA PAAD
Row counts (per table)	CLINICAL_IDENTIFIER	10	503	1000	459	522	99	155
	EXPERIMENT_RELATED_INFORMATION	10	517	1000	459	522	99	155
	BIOINFORMATICS_PROTOCOL_RELATED_INFORMATION	10	517	1000	459	522	99	155
	GENOMIC_ALTERATION	2733	29,279,631	842,199,347	361,933	318,947	229,525,363	56,159
	MICROSATELLITE_INSTABILITY	0	0	0	0	0	0	775
	CLINICAL_ANNOTATION	40	267	108	123	97	1	12
	QUALITY_CHECK	10	517	1000	0	0	0	0
Data volume (per database)		2MB	8.2GB	144.7GB	48.37MB	42.63MB	47.67GB	9.41MB

The databases are categorised into internal and demo database. The specifications of the database tables are informed in Table 1. This table presents row counts of each database table and data volumes of each database. The internal databases include 3 private datasets (cancer panel, leukemia and depression) and 2 public datasets (TCGA COAD and TCGA LUAD). The demo databases include 2 public datasets (1000 Genome Phase3 CEU and TCGA PAAD). * COAD is study abbreviation in the TCGA stands for Colon adenocarcinom a; LUAD for Lung adenocarcinoma.

#### How implementation of the cGDM enables interactive clinical decision support

**Figure 5:**
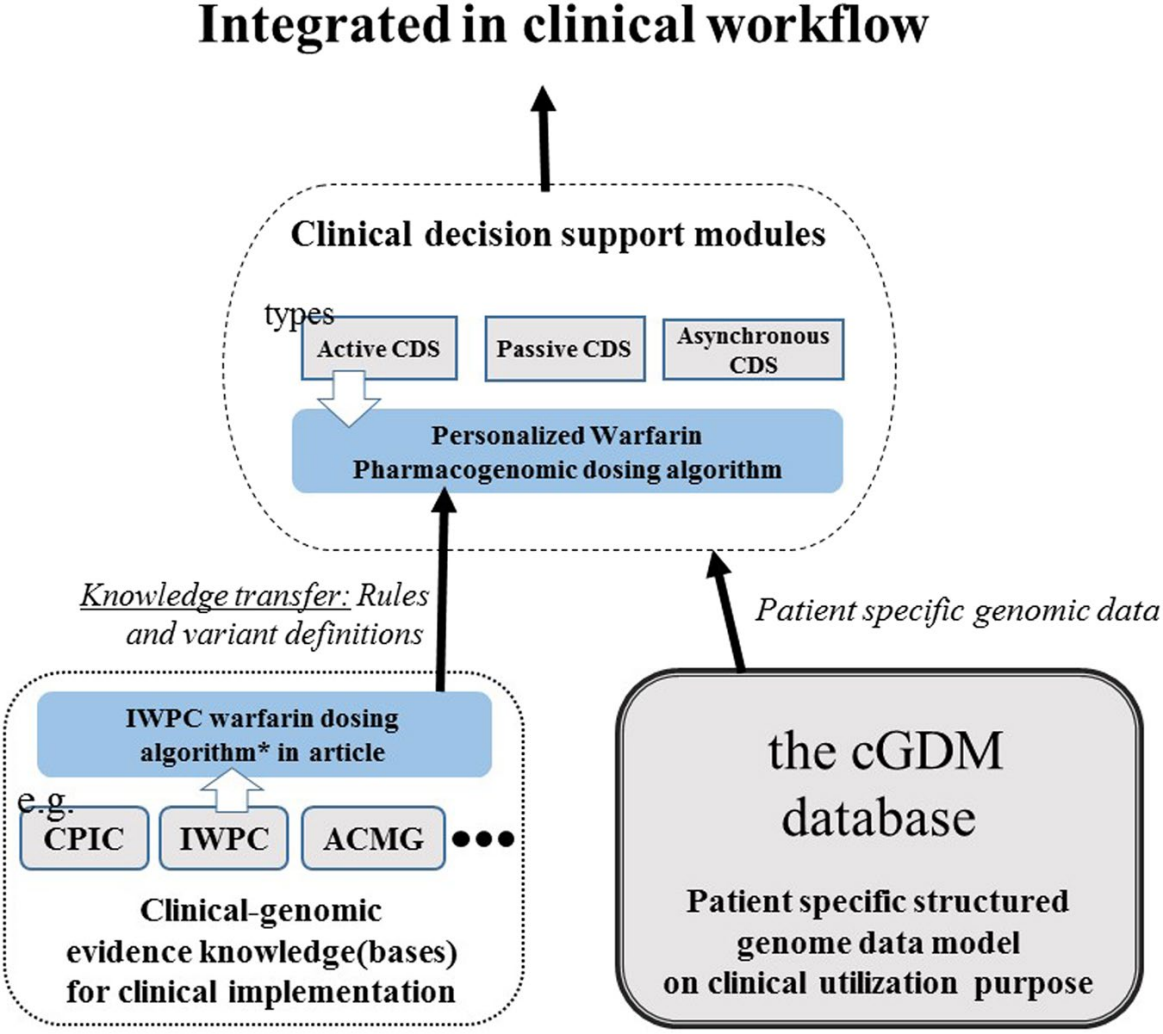
The conceptual map of a genomic decision support system based on the cGDM. While the accumulation of confirmatory knowledge could seem relatively slow compared to the speed of the vast discovery of the bioinformatics field, the benefits and impacts the two will have on patients when they are seamlessly connected are evident. The cGDM brings this process into computational space.

One of the major challenges of healthcare informatics is supporting clinicians who need to handle constantly evolving knowledge and inherently complex genomic data. Patient genomic data in a static document format or in structured model but in which has vague designation of the variant limits functionality of clinico-genomic information system^30^. The cGDM could address the issue by working as a data-level infrastructure for interactive clinical decision support along with external knowledge bases (Fig. 5). For the cGDM’s programmability test, we developed a pharmacogenomic clinical decision support function running on the cGDM database which reflects the knowledge of the IWPC warfarin dosing algorithm. The source code is freely available at https://github.com/SNUBI-HyojungKim/cGDM-Clinical-Genome-Data-Model. Supplementary Figure 3 illustrates both of logical information flow in back-end system and its appearance on the user interface.

## Discussion

The rapid accumulation of genomic information has led to a paradigm shift in medicine. However, significant barriers remain to overcome for the widespread clinical exploitation of this information. Through multi-disciplinary analysis and consideration of this phenomenon, we identified two main causes: first, reliability-related result variance among numerous pipelines and processes; and second, the unique data structure of genome information. Since these two causes have shared influences, an integrative solution is likely to be more effective than a point solution. Moreover, we foresee that GIS will become an essential component of an integrated clinical information system in the precision medicine era. In this context, this cGDM could serve as a genomic information representation scheme enabling the intellectual interaction between medical experts and informed decision making, ultimately contributing to the enhancement of personal genomic data utilisation at the point of care.

To ensure the convenient and appropriate clinical use of genomic data, medical informatics technology is needed as part of the infrastructure supporting the integration of clinic and genomic layers of information^31,32^ Given the multi-level and multi-dimensional nature of health, clinicians must perform decision-making for a given case based on a collection of segmented data representing a person’s health, including laboratory data, imaging, and observation data assessed by experts. Currently, a clinical information system is typically used as a core tool for supporting this knowledge in a management process. To broaden perspectives in the era of precision medicine, we propose a genome information system (GIS) as an integral component of an integrated clinical information system (Fig. 1).

The cGDM can serve as a data-level infrastructure for implementation of the GIS. When decision makers face unfamiliar health-status measurements, determining clinical significance and meaning is challenging^32,33^. The cGDM was designed to preserve genomic information at an appropriate information scale and granularity covering the procedural dimension, which is related to the confidence level as a clinical measurement for clinical application. The design of the cGDM allows processed genomic data for a general purpose to be stored and merged with existing clinical data, providing outputs in an interoperable data format. Likewise, sequencing analysis, data processing, and presentation of processed information can be managed in a form that can be explicitly confirmed. Once data are uploaded to the cGDM-based database, they serve as a supportive backbone for any downstream functional applications such as report generation or a clinical decision support system. (e.g. Supplementary Fig. 2; Fig. 4) To develop a system for the systematic management of genomic data, it is necessary to unify its data structure with that of other existing components of clinical information systems, ensuring sufficient reliability for identifying the original data generation process^34^.

Conventional systems have focussed on data structure unification issues first, to harmonise heterogeneous systems among separate institutions^35^. By contrast, our model was designed to achieve both clinico-genomic knowledge representation accompanied by traceability of the genomic data, to enable determination the clinical significance of a genomic test result provided to a clinician. To allow better assessment of the meaningfulness of genomic information, we defined the basis for each attribute and focused on designing an entity set that accurately represents the genomic data that are delivered to the target user, without information distortion. Furthermore, the cGDM is adaptable as a data-level extension to any existing information system, regardless of database system or application platform.

Accumulation of basic, translational, and regulatory science is a prerequisite to implementing personalised medicine in routine care^36^. As a basic science, bioinformatics has witnessed explosive and rapid progress since the completion of the Human Genome Project. In the context of regulatory science, there are currently several ongoing efforts within the bioinformatics and molecular biology domains^10–12^, with great maturation in the body of knowledge during the last decade, including principles and recommendations related to NGS technology. These efforts have focussed primarily on the standardisation of bioinformatics protocols and the file structures for intra- or interlaboratory communication.

Translational science represents the next challenge for the realisation of actual health promotion with personalised medicine^37^. In the context of clinico-genomics, translational approaches ultimately target the syntactic and semantic interoperability between genomics and clinical practice, to ensure business continuity in terms of knowledge management^37–39^. Previous approaches have stressed a need for structural transformation to overcome the currently low adaptation of genomic information for clinical decision-making. However, the other major cause, the knowledge gap, has yet to be seriously considered because the solution appears obvious: the education of medical experts in bioinformatics principles.

Nevertheless, this raises the question of the specific level of bioinformatics knowledge required in clinical practice. Our working group agreed that clinicians do not need to be bioinformatics experts to implement precision medicine. Preferably, the key is education on how to understand genomic data and confidence levels, and then be provided with sufficient information to make clinical decisions. Based on this perspective, we identified a previously unrecognised ambiguity related to the knowledge interplay between bioinformatics and medical practices (Fig. 3). Although the genome is the most concrete type of observational data representing an individual’s inheritance, the genomic information delivered to clinicians is rarely transformed to a human-readable form and is also rarely a direct representation of the genomic sequence. Instead, this information is more of an intellectual product, processed in a purpose-weighted result file structure. Thus, the question of reliability of the genomic information must be addressed before it is adopted by the physician, similar to other types of conventional observational data.

Considering the knowledge gap in this clinico-genomic context, unrecognised ambiguities may occur on each side. For example, when linking the outputs of bioinformatics to clinical fields, the indicator of information quality moves from internal consistency within the same protocol to external consistency between different protocols. Thus, to accomplish the final goal of precision medicine, more discussion is needed about how data will cross this intermediate space, then about how to best represent and deliver crossover information.

To best of our knowledge, the methodology proposed herein has not yet been applied in the field of genetic information processing. FMEA is the most commonly used methodology for determining reliability of manufacturing and design processes^15^–^19^. We perceive the result of genetic testing not as an output of static measurement, but rather as an output of an intellectual production process. When conducting bioinformatics analyses, there is no requirement for unification among the processes, since the internal consistency within each process guarantees scientific rigour. Moreover, the flexible data specifications used in the bioinformatics field have the advantage of supporting various research applications^7^, but that advantage becomes an obstacle to data integration for comprehensive clinical decision making. In addition, relevant external knowledge, tools, platforms, and analytical techniques cannot be unified because they are still under development. Considering this large interdisciplinary hyperspace, our approach aims to improve the quality of information delivery while responding to an enormous, growing body of knowledge that has yet to be integrated within its own basic-science field. Therefore, the FMEA was adopted to derive and clarify a set of metadata designed to prevent information from being distorted.

To facilitate the use of genomic test results in clinical practice, it is essential to integrate genomic data into clinical decision support systems regarding data volume and knowledge management^6,14,15,17^. Data modelling is the first and most crucial step in the multi-tiered design of information systems. The final product reliability, for example specific clinical decision support algorithms or integrated information systems, is hardly improved over the designed reliability on the lower level of architecture (data-level)^40^. This viewpoint was projected to the study design. An important consideration is that the analytic scheme presented here can help to enhance clinico-genomic understanding for experts on both the medical and bioinformatics sides of the workflow. (see Methods Section) Throughout the development of this method, we focussed on equally weighting the clinical perspective and bioinformatics process analysis in the context of business continuity, starting from our initial clinical intention through bioinformatics information processing by a knowledge-based protocol, finally offering a deliverable and interpretable form to the point-of-care clinician.

The methods, equipment, data processing and analytical techniques for extracting data from targets in nature will continue to evolve and accumulate. The cGDM was designed to be flexible and able to readily adapt to technological changes. However, an eventual failure in responding to these changes cannot be excluded and represents a potential limitation of this study.

Several standard models have been generated, based on differences in data scale and technical maturity, prior to the development of NGS technology. Thus, we have not considered multi-omics data. Focussing on NGS technology-based workflow helped us to determine an optimised information scale and granularity for the clinical level, and to design a model to generalise and process genomic data based on individual patients. The cGDM could be extended to be a part of technology-wide data model integration for multi-omics data management.

The data model proposed in this study aims to clarify blind points within the interdisciplinary genomic-clinical interface, connecting separated expertise within a single platform to provide a broad perspective that covers the information reliability required for clinical evidence. In particular, we have made a novel attempt to adopt the FMEA method for a systematic meta-level data design process. Future work will focus on the development of functional systems to conduct real-world validation, including a data-upload pipeline from processed genome data files, as well as a clinical decision support tools based on the cGDM. Results of this exercise are planned to be released in a further study.

## Methods

### Material: The production process of bringing genomic information to bedside care

Here, we define a genomic test as a series of team-based information production processes, in which the meaning of the information is expanded, represented, and reproduced by reference to an external knowledge base, rather than through direct extraction of inherent information. Despite the invariant nature of a personal genome, genomic information presented to a clinician may vary according to specific processing protocols adopted^7,25,26,41^. This variability raises reliability issues for the use of genomic test results as clinical evidence^42^.

As artefacts from production, genome information processed for clinical use may pose a likelihood of misinterpretation due to information distortion, omissions, and fragmented senses. Furthermore, information reliability is a critical factor determining the ability of clinicians to utilise the genomic information^43^. Thus, our approach in developing this cGDM for focussed on the multi-dimensional scope of information, including procedural factors, derived from NGS technology.

### FMEA: An attribute-clarified framework

FMEA is a systematic prospective risk factor analysis approach that predicts and prevents possible errors, improving quality across team-based processes^44^. When used for advanced investigation, the method has advantages enabling exploration of uncertain, unforeseen complex workflows at an early stage^45,46^. Since its introduction in 1963, broad subtype applications of FMEA have been performed in broad domains including reliability engineering^40,47^, behaviour modelling^48^, software engineering^49^, conceptual design^50^, and knowledge management and representation^51,52^. In particular, FMEA has been applied as a method of knowledge representation to extract process reliability related attributes and to structure and map entities and attributes^48,52–54^. In this study, the FMEA approach was adopted for workflow analysis and the attribute-extracting method.

#### The working group

A multidisciplinary expert team was formed from the areas of bioinformatics, medical informatics, and medicine. The participants included three bioinformaticians, two medical informaticians with clinical informatics and application expertise, and one medical doctor. The medical doctor has experience in both clinical practice and conducting translational research from the perspective of both biomedical science and clinical practice.

#### Workflow analysis

Over a period of nine months, process mapping, failure identification, and related attribute extraction were conducted using FMEA at over 18 team meetings. Structured data modelling for enhancement of data accessibility was then conducted using a logical data model, with the attribute set derived from the FMEA workflow diagram.

We chose the conventional FMEA workflow analysis^40,47^ and adapted it for cGDM development. Conventional FMEA consists of two main steps. First, the failure mode is identified through (1) assembling a multi-disciplinary team with at least one expert from each domain over the target production process, (2) combining components and process function in order to derive a workflow diagram, and (3) listing the modes that may lead to failure at each step. The second part involves modifying the process itself with consideration of priority, including (1) evaluating the severity and occurrence ranking of each failure mode and (2) proposing a modified workflow or audition guideline.

In this study, risk estimation and priority-scoring steps were not designed, since our purpose was to review the fragment of metadata composition that may cause unintended information distortion or misinterpretation.

### Logical data modelling

Data models are the basis of computation ability for intelligent information systems^55^. The database design process can generally be divided into logical and physical database design^56^. The physical data model requires a clear and specific description over logical design, which depends on the existing development environment. Thus, we designed this cGDM as a logical data model based on the FMEA results to support data-level integration with any existing clinical information systems.

Logical data modelling methods are comprised of abstraction and normalisation. Database abstraction refers to aggregation and generalisation that occur at the points of intersection^57^. We first abstracted the attributes derived from FMEA and expressed the factors corresponding to each step in the workflow. Then, normalisation was performed to prevent duplication and inconsistency of data elements considering their names, scale and relations.

### Demo datasets for the real-world data implementation

Two of representative public accessible datasets are selected for the development of the demo databases: The 1000 Genomes Project of the International Genome Sample Resource (IGSR) with population code “CEU” (Utah Residents with Northern and Western European Ancestry)^58^, the pancreatic cancer data from The Cancer Genome Atlas (TCGA_PAAD)^59^.

Collected datasets were VCF and MAF file format and Extract-Transformation-Load (ETL) process of the genomic data was performed by two bioinformaticians with Python 2.7.16. ANNOVAR 2016Oct24^60^ version was used as a clinical annotation tool for 1000 Genome Project CEU dataset. The result datasets were imported in a table within the MySQL server database by two medical informaticians. We ran the SQL scripts in MySQL 5.6.46 on a Server with 8GB of RAM and an NVIDIA tesla c1060 / Quad core CPU running run on CentOS Linux release 7.7.1908. The final outputs took the form of SQL tables and functions^61–63^.

## Supplementary information

Supplementary Information.

## Data Availability

The description of the internal datasets and demo datasets used in this work are summarized in Table 2. The internal datasets are available from the corresponding author on reasonable request. All public data utilized in this work are TCGA COAD, TCGA LUAD, TCGA PAAD (https://portal.gdc.cancer.gov/) and 1000 Genome Phase 3 CEU (https://www.internationalgenome.org/category/phase-3/). TCGA PAAD and 1000 Genome Phase 3 CEU are built in forms of the cGDM DB and shared as demo databases (available at https://github.com/SNUBI-HyojungKim/cGDM-Clinical-Genome-Data-Model). This repository contains Data-Definition-Language (DDL), two demo databases based on cGDM containing public data and a PGx CDS example source code in the case of IWPC warfarin dosing.
