# Supplementary material for: Clinical Genome Data Model (cGDM) provides Interactive Clinical Decision Support for Precision Medicine
Source: Sci Rep. 2020 Jan 29;10:1414. doi: 10.1038/s41598-020-58088-2 (PMC6989462; doi:10.1038/s41598-020-58088-2)
Supplement: Supplementary file 1 — Supplementary Information. [file 41598_2020_58088_MOESM1_ESM.docx]

**Clinical Genome Data Model (cGDM) provides Interactive Clinical Decision Support for Precision Medicine**

Authors: Hyo Jung Kim^1,#^, Hyeong Joon Kim^1,#^, Yoomi Park^1^, Woo-Seung Lee^1^, Younggyun Lim^1^, Ju Han Kim^1,^*

# These authors contributed equally to this work

^1^Seoul National University Biomedical Informatics (SNUBI), Division of Biomedical Informatics, Seoul National University College of Medicine, Seoul, Republic of Korea

**Contents**

Page

Supplementary Table S1. Table Specification of the cGDM implemented in RDBMS 2

Supplementary Figure S1. Entity-relationship diagram of the cGDM implemented in RDBMS 8

Supplementary Figure S2. Semantic search implementation based on the cGDM 9

Supplementary Figure S3. How implementation of the cGDM provides interactive clinical decision support in clinical information system 10

**Supplementary Table S1. Table Specification of the cGDM**

The logical entities and attributes expressed in Figure 3 were converted into physical entities and attributes. Here, we provided our physical data model as the following table. The required data type, description, and example value for each attribute defined are described. All of the logical entities and attributes in Figure 3 have been transformed and defined in the physical model presented here. So, by applying this sort of conversion to physical model, each researchers can construct a genomic database according to the environment of the existing information system.

| **CLINICAL IDENTIFIER Table specification** | | | | | | | | |
| --- | --- | --- | --- | --- | --- | --- | --- | --- |
| *#* | *Logical Name* | *Physical Name* | *PK* | *Required* | *Data Type* | *Description* | | *Example* |
| 1 | Subject Identifier | Subject_Identifier | PK | Yes | int(11) | Arbitrary person identifier defined in the cGDM database | | 1 |
| 2 | Patient Number | Patient_Number |  | Yes | varchar(20) | Patient number of existing HIS database used to link with the cGDM database | | 12345678 |
| 3 | Medical Institution Identifier | Institution_Identifier |  | Yes | varchar(20) | An abbreviation of the hospital name where the patient data linked with the cGDM database | | SNUH |
| 4 | Order Identifier | Order_Identifier |  | Yes | varchar(20) | Unique key value represents an order of existing HIS database used to link with the cGDM database | | 602489471 |
| 5 | Clinician Identifier | Clinicain_Identifier |  | Yes | varchar(20) | Unique key value represents a physician of existing HIS database used to link with the cGDM database | | A2068494 |
| 6 | Submission Date | Submission_Date |  | Yes | datetime | Date of the beginning of the data production period (e.g. ordered date) | | 2018-08-17 13:44 |
|  |  |  |  |  |  |  |  | |
| **EXPERIMENT RELATED INFORMATION Table specification** | | | | | | | | |
| *#* | *Logical Name* | *Physical Name* | *PK* | *Required* | *Data Type* | *Description* | | *Example* |
| 1 | Experiment Identifier | Experiment_Identifier | PK | Yes | int(11) | Arbitrary identifier of the experiment defined in the cGDM database | | 11 |
| 2 | Subject Identifier | Subject_Identifier | FK | Yes | int(11) | Arbitrary person identifier defined in the cGDM database | | 1 |
| 3 | Test Description | Test_Description |  | No | text | Detailed description for ordered test | |  |
| 4 | Type of sequencing | Sequencing_Type |  | Yes | varchar(50) | Library strategy for genome sequencing | | {WGS, WES, Targeted sequencing, etc.} |
| 5 | Platform technology | Platform_Technology |  | Yes | varchar(20) | The technology platform used to identify the variant | | NGS |
| 6 | Sequencer | Sequencer |  | Yes | varchar(50) | Sequencing equipment | | Illumina Hiseq 2500 |
| 7 | Sequencing Institution | Sequencing _Institution |  | Yes | varchar(50) | Name of sequencing institution | | SNUBI |
| 8 | Experimenter | Experimenter |  | Yes | varchar(50) | Name of the primary experimenter | | BJ Min |
| 9 | Collection Date | Collection_Date |  | Yes | datetime | Date of the sample collection | | 2018-09-03 11:00 |
|  |  |  |  |  |  |  | |  |
| **BIOINFORMATICS PROTOCOL RELATED INFORMATION Table specification** | | | | | | | | |
| *#* | *Logical Name* | *Physical Name* | *PK* | *Required* | *Data Type* | *Description* | | *Example* |
| 1 | Bioinformatics Protocol Identifier | BI_Protocol_Identifier | PK | Yes | int(11) | Arbitrary identifier of the bioinformatics protocol defined in the cGDM database | | 121 |
| 2 | Experiment Identifier | Experiment_Identifier | FK | Yes | int(11) | Arbitrary identifier of the experiment defined in the cGDM database | | 11 |
| 3 | Pipeline Name | Pipeline_Name |  | Yes | varchar(50) | Name of the pipeline | | SNUBI WXS data pipeline |
| 4 | Step (of the pipeline) | Step |  | Yes | int(3) | The order in which the steps are executed | | 1 |
| 5 | Tool (of the pipeline) | Tool |  | Yes | varchar(50) | Procedure description | | (alignment, sort, deduplication, variant calling, etc.} |
| 6 | Parameter (of the pipeline) | Parameter |  | Yes | varchar(50) | The name of tools | | GATK |
| 7 | Datasource origin (used in the pipeline) | Datasource_Origin |  | Yes | varchar(50) | The version of tools | | v2.5-2 |
| 8 | Datasource version (used in the pipeline) | Datasource_Version |  | No | varchar(50) | Preset parameters used for the step | | stand_call_conf=30,stand_emit_conf=10 |
| 9 | Datasource Build (used in the pipeline) | Datasource_Build |  | No | varchar(50) | The source of databases | | 1kG, Mills, dbSNP137 |
| 10 | Analytics Institution | Analytics_Institution |  | Yes | varchar(50) | Name of the bioinformatics analytics institution | | SNUBI |
| 11 | Bioinformatician | Bioinformatician |  | Yes | varchar(50) | Name of the primary bioinformatician | | YM Park |
| 12 | Received Date | Received_Date |  | Yes | datetime | Date of the raw data file (eg. BAM file) received | | 2018-09-15 17:35 |
| 13 | Documentation Date | Documentation_Date |  | Yes | datetime | Date of the processed data stored in the cGDM database | | 2018-09-22 11:22 |
|  |  |  |  |  |  |  |  | |
| **QUALITY CHECK Table specification** | | | | | | | | |
| *#* | *Logical Name* | *Physical Name* | *PK* | *Required* | *Data Type* | *Description* | | *Example* |
| 1 | Quality Check Identifier | QC_Identifier | PK | Yes | int(11) | Arbitrary identifier of the quality check matrix in the cGDM database | | 123 |
| 2 | Bioinformatics Protocol Identifier | BI_Protocol_Identifier | FK | Yes | int(11) | Arbitrary identifier of the bioinformatics protocol in the cGDM database | | 121 |
| 3 | Total Reads | Total_Reads |  | Yes | bigint | Total number of reads | | 100720000 |
| 4 | Total Aligned Reads | Total_Aligned_Reads |  | No | bigint | Total number of aligned reads | | 99168912 |
| 5 | % Reads Aligned | Reads_Aligned_Percent |  | No | float | Percentage of reads aligned | | 98.46 ( = 4/3) |
| 6 | Total Bases | Total_Bases |  | No | bigint | Total number of bases | | 7260000 |
| 7 | Total Mapped Bases | Mapped_Bases |  | No | bigint | Total number of mapped bases | | 7050000 |
| 8 | Average on target depth | Depth_Mean |  | No | float | Mean on target depth | | 71.94 |
| 9 | Standard deviation on target depth | Depth_SD |  | No | float | Standard deviation of on target depth | | 16.54 |
| 10 | On Target Bases | Target_Bases |  | No | bigint | On target bases | | 2640000 |
|  |  |  |  |  |  |  |  | |
| **GENOMIC ALTERATION Table specification** | | | | | | | | |
| *#* | *Logical Name* | *Physical Name* | *PK* | *Required* | *Data Type* | *Description* | | *Example* |
| 1 | Genomic Alteration Identifier | Genomic_Alteration_Identifier | PK | Yes | int(11) | Arbitrary identifier of the genomic alteration defined in the cGDM database | | 14009 |
| 2 | Bioinformatics Protocol Identifier | BI_Protocol_Identifier | FK | Yes | int(11) | Arbitrary identifier of the bioinformatics protocol defined in the cGDM database | | 121 |
| 3 | Position | Position |  | Yes | varchar(255) | The genomic position where the alteration occurs | | 180888597 |
| 4 | Reference allele | Reference_Allele |  | Yes | varchar(255) | The base found in the reference genome | | A |
| 5 | Alternative allele | Alternative_Allele |  | Yes | varchar(255) | Any base other than the reference | | T |
| 6 | Chromosome | Chromosome |  | Yes | varchar(2) | The chromosome where the alteration occurs | | 7 |
| 7 | Cytogenetic location | Cytogenetic_Location |  | No | text | Cytogenetic band that the location of the alteration maps to | | 17q12 |
| 8 | Codon | Codon |  | No | text | The codon where the alteration is identified | | 12 |
| 9 | Exon | Exon |  | No | varchar(10) | The exonic location where the alteration is identified | | 19 |
| 10 | HGVS genomic change | HGVS_Genomic_Change |  | Yes | text | Description of the nucleotide change for a genomic sequence  (supplied by HGVS) | | NG_007873.3:g.176429T>A |
| 11 | HGVS coding change | HGVS_Coding_Change |  | No | text | Description of the nucleotide change for a coding DNA sequence  (supplied by HGVS) | | NM_004333.4:c.1799T>A |
| 12 | HGVS protein change | HGVS_Protein_Change |  | No | text | Description of the nucleotide change for a protein sequence  (supplied by HGVS) | | NP_004324.2:p.Val600Glu |
| 13 | HGVS version | HGVS_Version |  | Yes | varchar(20) | The version number of HGVS | | HGVS version 15.11 |
| 14 | dbSNP Identification Number | dbSNP_ID |  | No | varchar(20) | The identification tag (supplied by NCBI dbSNP) | | rs56046546 |
| 15 | dbVar Identification Number | dbVar_ID |  | No | varchar(20) | The identification tag (supplied by NCBI dbVar) | | nsv1123397 |
| 16 | Genome build | Genome_Build |  | No | varchar(20) | Genomic coordinates of the reference | | GRCh37/hg19 |
| 17 | Genomic source | Genomic_Source |  | Yes | varchar(10) | Class of genomic source | | {Somatic, Germline, Unknown, etc.} |
| 18 | HGNC gene symbol | HGNC_Gene_Symbol |  | No | varchar(20) | The official gene symbol approved by the HGNC | | ALK, JMJD7-PAL2G4B |
| 19 | Entrez gene ID | Entrez_ID |  | No | integer | Entrez Gene ID (supplied by NCBI) | | 238 |
| 20 | Ensembl gene ID | Ensembl_ID |  | No | char(15) | Ensembl Gene ID (supplied by Ensembl) | | ENSG00000171094 |
| 21 | Genotype | Genotype |  | No | char(3) | Allelic state of the given variant | | 0\|1, 0\|0, .\|., etc |
| 22 | clinVar Variation Identification Number | clinVar_Variant_ID |  | No | varchar(20) | The identification tag (supplied by clinVar) | | 188275 |
| 23 | COSMIC Identification Number | COSMIC_ID |  | No | varchar(10) | The identification tag (supplied by COSMIC) | | COSM476 |
| 24 | Molecular Effects | Molecular_Effect |  | No | varchar(50) | Effects of mutations on protein function | | {Missense, Nonsense, Frameshift, Promoter, etc} |
| 25 | Variant type | Variant_Type |  | Yes | varchar(20) | The type of variant in a sequence of DNA | | {Substitution, Deletion, Duplication, Insertion, InDel, Inversion, Conversion, etc.} |
| 26 | Functional Domain | Functional_Domain |  | No | varchar(50) | The functional domain where the alteration occurs | | ATP-binging domain |
|  |  |  |  |  |  |  |  | |
| **CLINICAL ANNOTATION Table specification** | | | | | | | | |
| *#* | *Logical Name* | *Physical Name* | *PK* | *Required* | *Data Type* | *Description* | | *Example* |
| 1 | Clinical Annotation Identifier | Clinical_Annotation_Identifier | PK | Yes | int(11) | Arbitrary identifier of the clinical annotation defined in the cGDM database | | 22 |
| 2 | Genomic Alteration Identifier | Genomic_Alteration_Identifier | FK | Yes | int(11) | Arbitrary identifier of the genomic alteration defined in the cGDM database | | 14009 |
| 3 | Biomarker Datasource | Biomarker_Datasource |  | Yes | varchar(255) | Name of datasource for biomarkers of genomic data | | ACMG actionable genes |
| 4 | Biomarker Name | Biomarker_Name |  | Yes | varchar(50) | Name of predictive indicator from biomarker datasource | | EGFR Exon 19 Deletion |
|  |  |  |  |  |  |  | |  |
| **MICROSATELLITE INSTABILITY Table specification** | | | | | | | | |
| *#* | *Logical Name* | *Physical Name* | *PK* | *Required* | *Data Type* | *Description* | | *Example* |
| 1 | Microsatellite Instability Identifier | MSI_Identifier | PK | Yes | int(11) | Arbitrary identifier of microsatellite instability defined in the cGDM database | | 14 |
| 2 | Bioinformatics Protocol Identifier | BI_Protocol_Identifier | FK | Yes | int(11) | Arbitrary identifier of the bioinformatics protocol defined in the cGDM database | | 121 |
| 3 | MSI phenotype | MSI_Phenotype |  | Yes | varchar(50) | Distinct phenotype of the microsatellite instability | | {Microsatellite Stable (MSS), MSI-Low (MSI-L), MSI-High (MSI-H), Indeterminate MSI} |
| 4 | MSI marker name | MSI_Marker_Name |  | Yes | varchar(20) | Name of the MSI marker | | BAT26 |
| 5 | MSI marker status | MSI_Marker_Status |  | Yes | varchar(20) | Determined MSI status | | Positive |

**Supplementary Figure S1. Entity-relationship diagram of the cGDM implemented in RDBMS**

**
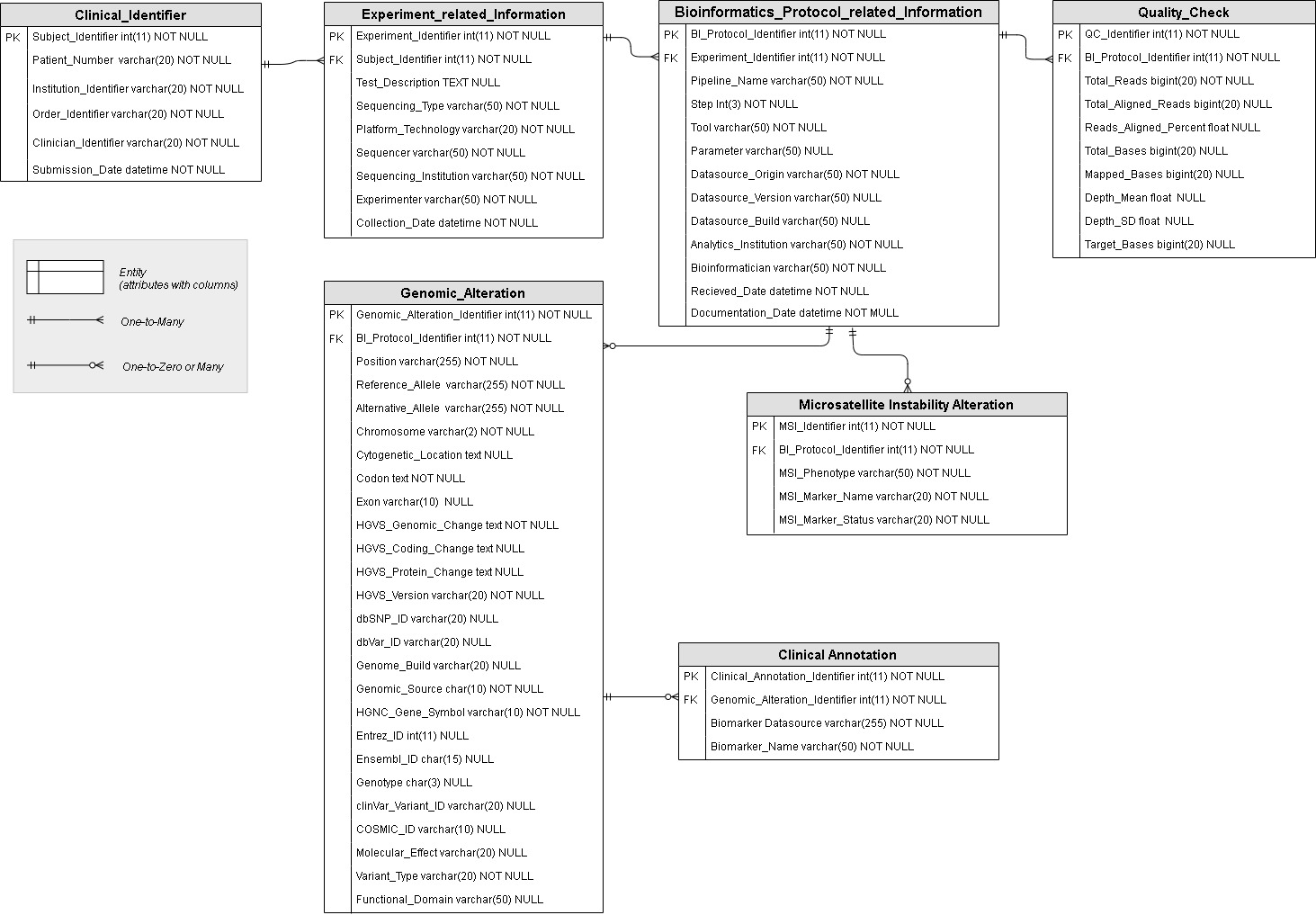
**The entity-relation for the physical model as a diagram (ERD) was presented based on the table shown in Supplementary Table 1. The diagram shows the entities and the attributes that describes the entity, and the relationship between the entities is also defined.

**Supplementary Figure S2. Semantic search implementation based on the cGDM**

Even if the user does not know all the nomenclature or metadata relevant to the genomic data to be searched, search function based on the cGDM can uses information entered in the search fields in order to derive an extended search result. Through the generated SQL syntax, the user can determine which genomic metadata (such as chromosome and position, genome build version, HGVS ID) can be associated and extended to the outcome of the patient's data. In addition to the attributes "Biomarker" and "HGVS ID" presented in the example, multiple data queries can be made with a single attribute or combination of attributes presented in the cGDM. Therefore, by using these user interfaces with the data model, it is possible to trace and verify whether the queried genomic data of the patient represent more reliable information.

**
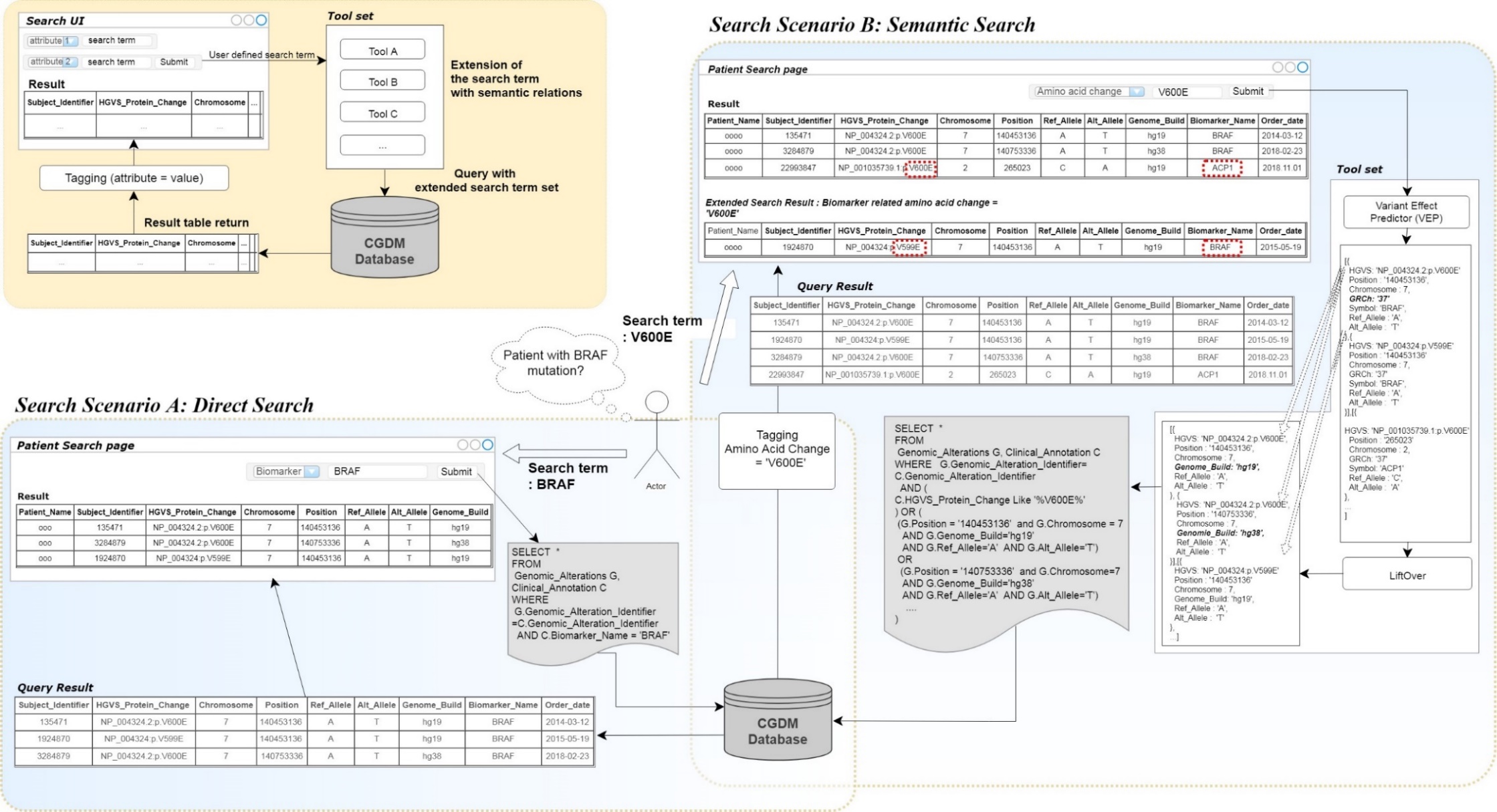
**

**Supplementary Figure S3. How implementation of the cGDM provides interactive clinical decision support in clinical information system**

A: When a doctor enters a prescription, a dataset for the prescription is generated and transmitted for storage. B: The dataset is passed along to the CDS module to search for the relevant knowledge base in accordance with a predefined set of rules. In this case, we internalized the systematic reference to the IWPC algorithm* integrated with the cGDM database. C: The PGx CDS module based on the cGDM selects the patient specific warfarin dosing related variant information which matches the IWPC algorithm in real-time. The cGDM produces an effect as knowledge representation backbone as well as genomic data storage scheme in the process. (e.g. Expression converted from input variables(Ci) to output variable(Co) for further processing.) D: The recommendation, which personalized dosing results from the IWPC warfarin PGx estimation based on both clinical and genomic factors, are delivered to the prescriber. Trackable links for each origin of the used genomic data and evidence in the algorithm are also provided.

**
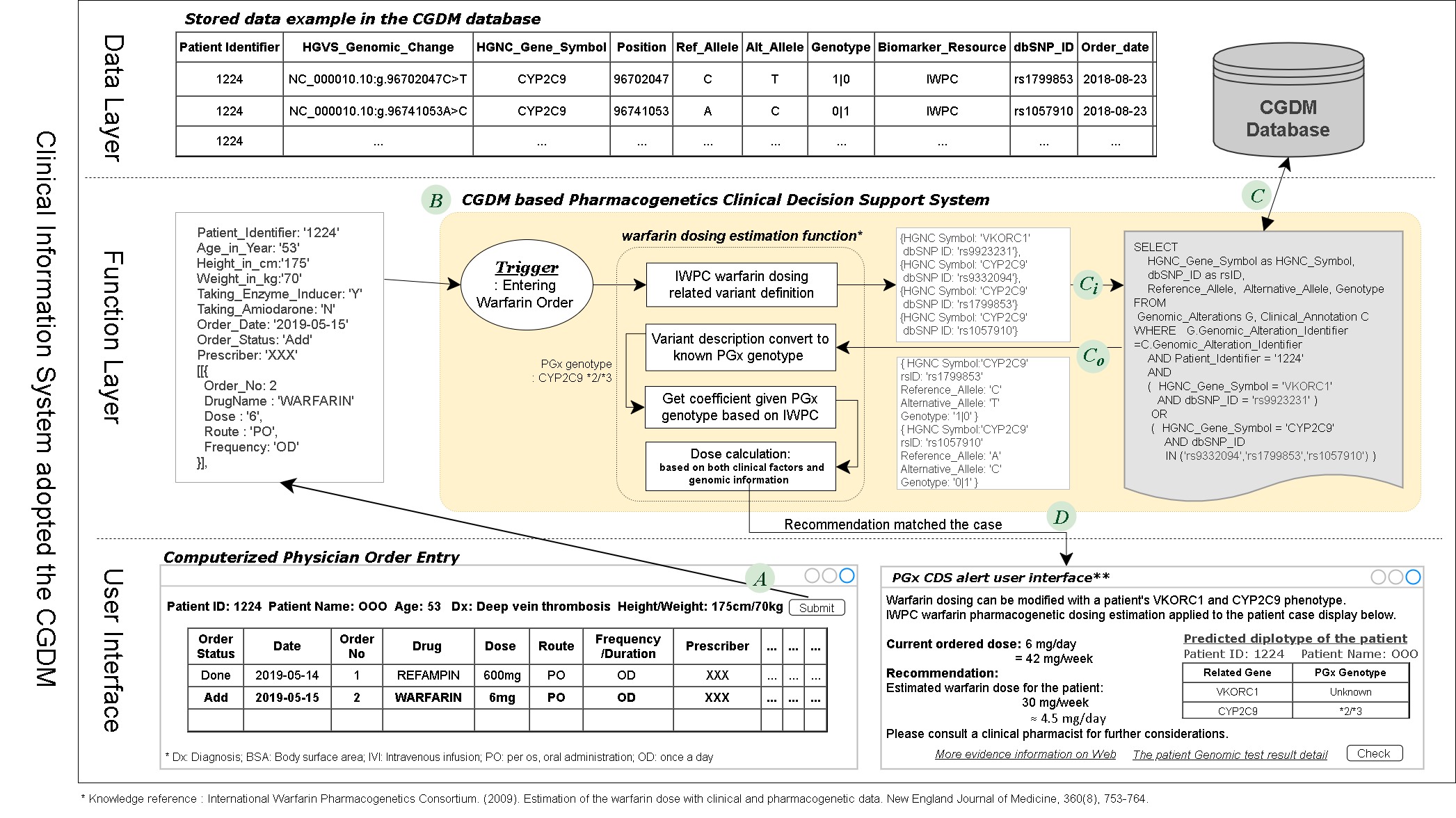
**

* International Warfarin Pharmacogenetics Consortium. (2009). Estimation of the warfarin dose with clinical and pharmacogenetic data. New England Journal of Medicine, 360(8), 753-764.

** Demo database and source of the software is also accessible at: https://github.com/SNUBI-HyojungKim/cGDM-Clinical-Genome-Data-Model/
